# Genome Sequence of a SARS-CoV-2 P.1 Variant of Concern (20J/501Y.V3) from Bangladesh

**DOI:** 10.1128/MRA.00524-21

**Published:** 2021-07-08

**Authors:** M. Murshed Hasan Sarkar, Mohammad Fazle Alam Rabbi, Shahina Akter, Tanjina Akhtar Banu, Barna Goswami, Iffat Jahan, M. Saddam Hossain, Eshrar Osman, Mohammad Samir Uzzaman, M. Ahashan Habib, Abu Sayeed Mohammad Mahmud, Firoz Kabir, Kazi Nadim Hasan, M. Mizanur Rahman, M. Abdul Khaleque, Sharif Akhteruzzaman, M. Salim Khan

**Affiliations:** a Bangladesh Council of Scientific & Industrial Research (BCSIR), Dhaka, Bangladesh; b DNA Solution Ltd., Dhaka, Bangladesh; c Department of Soil, Water and Environment, University of Dhaka, Dhaka, Bangladesh; d SciTech Consulting and Solutions, Dhaka, Bangladesh; e Department of Biochemistry and Microbiology, School of Health and Life Sciences, North South University, Dhaka, Bangladesh; f NIPRO JMI Pharma, Dhaka, Bangladesh; g Department of Genetic Engineering and Biotechnology, University of Dhaka, Dhaka, Bangladesh; DOE Joint Genome Institute

## Abstract

This study reports the coding-complete genome sequence, with variant identifications and phylogenetic analysis, of a severe acute respiratory syndrome coronavirus 2 (SARS-CoV-2) P.1 variant (20J/501Y.V3), obtained from an oropharyngeal swab specimen from a female Bangladeshi patient diagnosed with coronavirus disease 2019 (COVID-19) with no travel history.

## ANNOUNCEMENT

Since March 2021, the confirmed number of positive COVID-19 cases and the associated death toll have been increasing in Bangladesh compared to late 2020 and early 2021 national surveillance data (1). Severe acute respiratory syndrome coronavirus 2 (SARS-CoV-2) is the etiological agent of COVID-19 and belongs to the family *Coronaviridae* and the genus *Betacoronavirus*. Variants of the virus have been reported already, among them variants of great concern (B.1.1.7, 20I/501Y.V1, and B.1.351 [20H/501Y.V2]) due to their possible increased transmissibility and demonstrated resistance to neutralizing antibody therapy targeted at the N-terminal domain (NTD) or the receptor-binding domain (RBD) (2). Variant P.1 (20J/501Y.V3) is also a variant of concern and shares three substitution mutations (K417T, E484K, and N501Y) at the RBD site similar to the B.1.351 (20H/501Y.V2) variant. Here, we report the coding-complete genome sequence of a SARS-CoV-2 P.1 variant (20J/501Y.V3) from Bangladesh. Ethical approval was received from the National Institute of Laboratory Medicine and Referral Center (NILMRC) of the Bangladesh government to sequence strains from human subjects at the Genomic Research Laboratory of BCSIR, where this study was carried out.

A patient with a suspected COVID-19 case with a fever and respirational distress visited the outdoor patient unit of DNA Solution Ltd. on 18 February 2021. The 37-year-old female patient had a history of asthma with no other known comorbidities. The patient had no travel history but had come into contact with a traveler from South Africa. An oropharyngeal swab specimen was collected and was tested for SARS-CoV-2 RNA using a commercial one-step COVID-19 real-time PCR kit (Sansure Biotech Inc., Changsha, China) following the manufacturer’s instructions. The sample tested positive for SARS-CoV-2 RNA with a cycle threshold (*C_T_*) value of less than 30. The sample was then sent out for sequencing at the Genomic Research Laboratory of the Bangladesh Council of Scientific and Industrial Research (BCSIR). There, viral RNA was extracted using the ReliaPrep viral total nucleic acid purification kit (Promega, USA). Viral cDNA libraries were prepared using the RNA prep with enrichment (L) tagmentation kit according to the manufacturer's protocol (Illumina, CA, USA). After tagmentation and amplification, the sample was enriched using the Illumina respiratory virus oligonucleotide panel version 2, and a library was generated. The library was sequenced using the MiniSeq sequencing system with an output of 2 × 74-bp paired-end reads. A total of 4,831,986 reads were generated, of which 4,753,260 (98.37%) belonged to the SARS-CoV-2 genome. The sequenced reads of SARS-CoV-2 genomes were assembled using Illumina DRAGEN RNA Pathogen Detection software version 3.5.14 with default settings. The sequencing data were deposited in the NCBI GenBank database (accession number MZ020420).

**FIG 1:**
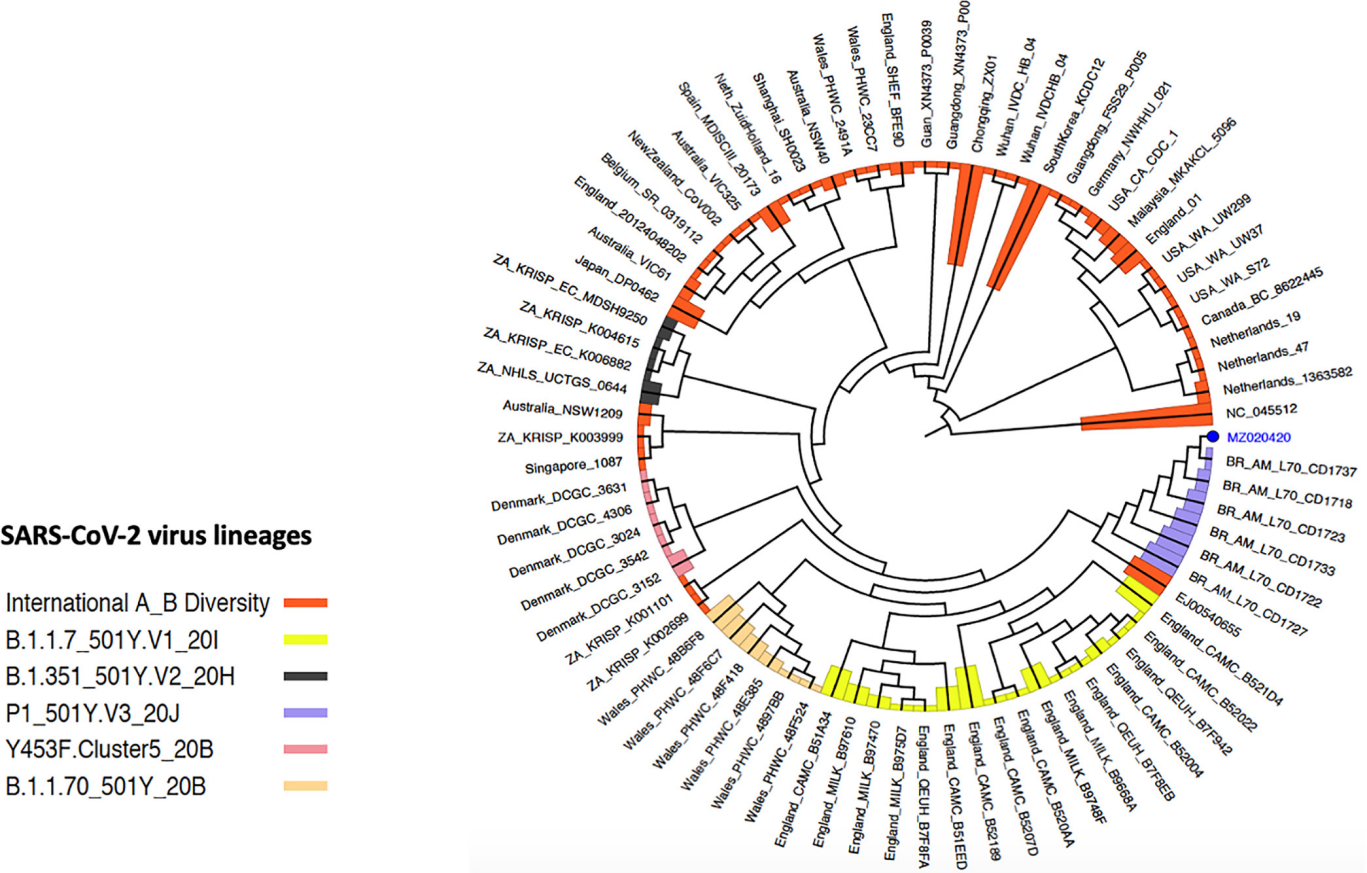
Schematic polar diagram displaying the phylogenetic tree of the strain found under the GenBank accession number MZ020420. The FASTA file was uploaded to the Genome Detective Coronavirus Typing Tool version 1.17. The toolkit uses BLAST and phylogenetic methods to assign a lineage to the virus sequence according to a method described by Cleemput et al. (7). The genome of interest is of the P.1 (20J/501Y.V3) lineage.

Further, genome assembly of the raw data was performed using the assembly toolkit in the EzCOVID19 cloud service (3), provided on the EzBioCloud website (4). The assembly was performed by aligning reads to the Wuhan predefined reference genome (GenBank accession number NC_045512.2). The genome sequence consisted of 29,789 nucleotides and covered 99.62% of the SARS-CoV-2 Wuhan reference genome (NC_045512.2), with a GC content of 38%. A consensus genome was created to retain the unique variations of the raw data using the EzCOVID19 cloud service (3, 5). The consensus genome was then compared against the same reference genome to calculate single nucleotide variations (SNVs) and positions. The SNVs were compared against GISAID clade variation markers (6). The genome presented here belonged to the P.1 (20J/501Y.V3) lineage of clade GR. This was further verified by analyzing the raw data using the default parameters of the Coronavirus Typing Tool offered by Genome Detective (7) (Fig. 1).

### Data availability.

The SARS-CoV-2 sequence was deposited in GenBank under the accession number MZ020420. The raw sequence reads have been deposited in the NCBI Sequence Read Archive (SRA) under the accession number SRX11071271 and the BioProject accession number PRJNA733209.
